# Development of a Novel Nutrition-Related Multivariate Biomarker for Mild Cognitive Impairment Based on the Plasma Free Amino Acid Profile

**DOI:** 10.3390/nu14030637

**Published:** 2022-02-01

**Authors:** Takeshi Ikeuchi, Yuki Yano, Wataru Sato, Fumiyoshi Morikawa, Shuta Toru, Chika Nishimura, Nobuhiko Miyazawa, Yasuko Kuroha, Ryoko Koike, Shin Tanaka, Kumiko Utsumi, Kensaku Kasuga, Takayoshi Tokutake, Kenjiro Ono, Satoshi Yano, Satoshi Naruse, Ryuji Yajima, Tadanori Hamano, Yuri Yokoyama, Akihiko Kitamura, Eiji Kaneko, Minoru Yamakado, Kenji Nagao

**Affiliations:** 1 Department of Molecular Genetics, Brain Research Institute, Niigata University, Niigata 951-8585, Japan; ken39@bri.niigata-u.ac.jp (K.K.); tokutaketaka@yahoo.co.jp (T.T.); 2 Research Institute for Bioscience Products & Fine Chemicals, Ajinomoto Co., Inc., Kawasaki City 210-8681, Japan; yuki.yano.ux5@asv.ajinomoto.com (Y.Y.); wataru.sato.m7h@asv.ajinomoto.com (W.S.); 3 Department of Psychiatry, Asahikawa Keisenkai Hospital, Asahikawa 078-8208, Japan; f.morikawa@keisenkai.or.jp; 4 Department of Neurology, Nitobe Memorial Nakano General Hospital, Tokyo 164-0011, Japan; shuta-toru@nakanosogo.or.jp; 5 Kurumi Clinic, Tokyo 156-0041, Japan; cnishi@dd.iij4u.or.jp; 6 Department of Neurosurgery, Kofu Neurosurgical Hospital, Yamanashi 400-0805, Japan; nobu_miya9311@yahoo.co.jp; 7 Department of Neurology, Nishiniigata Chuo Hospital, Niigata 950-2085, Japan; kuroha.yasuko.yu@mail.hosp.go.jp (Y.K.); ryoko.koike@aiko.or.jp (R.K.); 8 Mishima Hospital, Niigata 940-2302, Japan; shin_tanaka39@hotmail.com; 9 Department of Psychiatry, Sunagawa City Medical Center, Sunagawa 073-0196, Japan; utsumimomo@s9.dion.ne.jp; 10 Department of Neurology, Brain Research Institute, Niigata University, Niigata 951-8585, Japan; 11 Division of Neurology, Department of Medicine, Showa University School of Medicine, Tokyo 142-8666, Japan; onoken@med.kanazawa-u.ac.jp (K.O.); yanoyanoyano10@yahoo.co.jp (S.Y.); 12 Department of Neurology, Midori Hospital, Niigata 950-0983, Japan; naruse@midori-gr.jp (S.N.); yajima.ryuji@midori-gr.jp (R.Y.); 13 Second Department of Internal Medicine, Faculty of Medical Sciences, University of Fukui, Fukui 910-1193, Japan; hamano@u-fukui.ac.jp; 14 Research Team for Social Participation and Community Health, Tokyo Metropolitan Institute of Gerontology, Tokyo 173-0015, Japan; yokoyama@tmig.or.jp (Y.Y.); kitamura@tmig.or.jp (A.K.); 15 Institute of Education, Tokyo Medical and Dental University, Tokyo 113-8519, Japan; eiji.vasc@tmd.ac.jp; 16 Department of Nursing, Ashikaga University, Ashikaga 326-0808, Japan; yamakado.minoru@v90.ashitech.ac.jp

**Keywords:** multicenter clinical study, protein malnutrition, biomarker discovery, *APOE*, MMSE

## Abstract

Nutritional epidemiology has shown the importance of protein intake for maintaining brain function in the elderly population. Mild cognitive impairment (MCI) may be associated with malnutrition, especially protein intake. We explored blood-based biomarkers linking protein nutritional status with MCI in a multicenter study. In total, 219 individuals with MCI (79.5 ± 5.7 year) from 10 institutions and 220 individuals who were cognitively normal (CN, 76.3 ± 6.6 year) in four different cities in Japan were recruited. They were divided into the training (120 MCI and 120 CN) and validation (99 MCI and 100 CN) groups. A model involving concentrations of PFAAs and albumin to discriminate MCI from CN individuals was constructed by multivariate logistic regression analysis in the training dataset, and the performance was evaluated in the validation dataset. The concentrations of some essential amino acids and albumin were significantly lower in MCI group than CN group. An index incorporating albumin and PFAA discriminated MCI from CN participants with the AUC of 0.705 (95% CI: 0.632–0.778), and the sensitivities at specificities of 90% and 60% were 25.3% and 76.8%, respectively. No significant association with BMI or *APOE* status was observed. This cross-sectional study suggests that the biomarker changes in MCI group may be associated with protein nutrition.

## 1. Introduction

Alzheimer’s disease (AD) is a neurodegenerative condition that is highly prevalent in old age [1] and has a significant socioeconomic impact [2]. For example, Japan has a super-aging society, and the estimated population with dementia will rise from 4.4 million in 2018 to over 7 million by 2025, which will represent one in five people over 65 years old [3]. As the care for patients with diseases that affect cognitive function require the support of multiple stakeholders, they impose a substantial socioeconomic burden [2].

Pathological changes, such as the accumulations of amyloid-β (Aβ) and hyperphosphorylated tau in the brain, occur more than two decades before the appearance of cognitive impairment [1]. Even if the onset of cognitive impairments could be delayed by a novel medication that could prevent such accumulation for decades, it is economically challenging to continue a treatment intervention for this long from the preclinical period. In this context, early risk detection and the modification of risk factors for AD, such as diet and lifestyle, could be a realistic strategy for AD prevention. Although the causes of dementia are complex and multifactorial, it has been estimated that up to one-third of dementia cases may be prevented [4]. In the long-term randomized controlled trial called the Finnish Geriatric Intervention Study to Prevent Cognitive Impairment and Disability (FINGER), a multidomain lifestyle-based intervention ameliorating vascular and lifestyle-related risk factors was found to preserve cognitive function and reduce the risk of cognitive decline among older adults with increased risk of dementia [5].

A recent paper observing 3632 participants in the Framingham study reported that low body mass index (BMI), which is a simple index of weight-for-height measurements and commonly used to classify underweight, overweight, and obesity in adults, increases the risk of dementia in old age (>50 years) [6]. In this context, nutrition is one of the key modifiable risk factors for preventing dementia. The presence of malnutrition can lead to prefrailty/frailty, and the presence of prefrailty/frailty can lead to malnutrition, forming a vicious cycle that results in poor health outcomes [7]. Malnutrition is associated with cognitive impairment and functional loss, and it is also known that an inadequate nutritional status predisposes individuals to cognitive frailty. To date, nutritional epidemiology has shown the importance of protein intake for maintaining brain function in the elderly population. Compared with healthy elderly individuals, patients with dementia have significantly lower protein intake [8,9,10]. The level of protein intake in elderly people is positively associated with memory function [11,12]. Elderly people with high protein intake levels have a lower risk of mild cognitive impairment (MCI) [13], as well as lower levels of Aβ accumulation in the brain [14]. Furthermore, when discussed in relation to genetic risk factors, the Rotterdam Study reported that a healthy lifestyle including diet reduced the risk of dementia in individuals with genotypes that did not carry apolipoprotein E (*APOE*) ε4 allele [15]. Therefore, it would be useful to have a biomarker for nutritional status, which is associated with cognitive decline, and also to clarify its relationship with *APOE* ε4 allele status.

We thus focused on the concentrations of amino acids and albumin in the blood, as these are possible biomarkers that could link protein nutrition status and cognitive decline. Albumin is the most commonly used marker of protein nutritional status, and albumin concentration is reduced by malnutrition and inflammation [16]. Additionally, in recent years, attention has been focused on the usefulness of plasma free amino acids (PFAAs) as biomarkers. The assessment of PFAAs has the advantages of reproducibility, accuracy, and high-throughput screening capability [17,18], and reference intervals have already been established [19]. Therefore, we conducted a multicenter clinical study with 219 elderly individuals with MCI and 220 cognitively normal (CN) individuals to develop and validate an index incorporating PFAAs and albumin as biomarkers.

## 2. Materials and Methods

### 2.1. Ethics Statement

This study was conducted in accordance with the Declaration of Helsinki. The study protocol was approved by the ethics committee of each institution, including Niigata University and Ajinomoto Co., Inc., and was registered with the University Hospital Medical Information Network Clinical Trials Registry with the number UMIN000021965. All participants gave written informed consent before participating in this study. All clinical information was anonymized before data analysis.

### 2.2. Participants

MCI individuals (*n* = 219) were recruited between 2016 and 2020 from the following 10 institutions: Niigata University Medical and Dental Hospital, Asahikawa Keisenkai Hospital, Nitobe Memorial Nakano General Hospital, Kurumi Clinic, Kofu Neurosurgical Hospital, Nishiniigata Chuo Hospital, Mishima Hospital, Sunagawa City Medical Center, Showa University Hospital, and Midori Hospital. CN control participants (*n* = 220) who underwent comprehensive health examinations were recruited from community-dwelling adults in four different cities in Japan (Hatoyama town, Mitsuke city, Ashikaga city, and Kawasaki city), and CN individuals who visited Sunagawa City Medical Center.

### 2.3. Cognitive Assessment and Inclusion and Exclusion Criteria

To assess the cognitive function of the participants, neuropsychological assessments were carried out. The Mini Mental State Examination (MMSE) was employed to assess general cognitive function. The Wechsler Memory Scale-Revised Logical Memory II (WMS-R LM II) subtest or Clinical Dementia Rating (CDR) was employed to assess the cognitive function and the severity of dementia. To assess the degree of depression, the Geriatric Depression Scale-15 (GDS-15) was employed.

**Figure 1:**
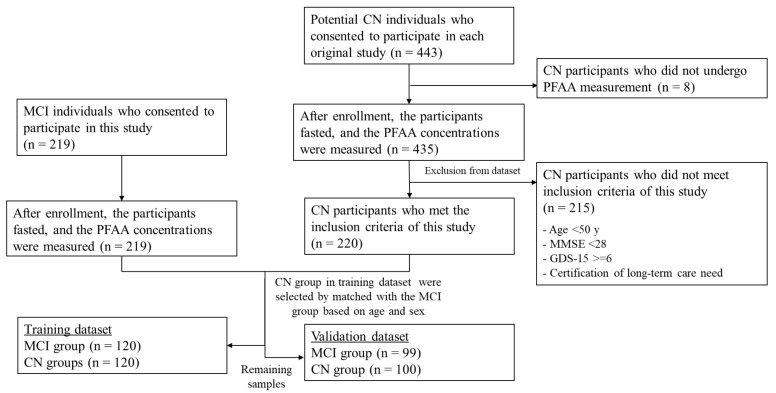
Summary of study design.

Individuals who met all the following criteria were included. The common criteria for MCI and CN participants were as follows: (1) aged 50 years or older; (2) not diagnosed with dementia; (3) living independently; (4) able to undergo neuropsychological tests, with an MMSE score of 24–30 points; and (5) no depression and a GDS-15 score less than 6 points. In addition, if either of the following was applicable, the participant was included in the MCI group: (1) WMS-R LM II score of 11 points or less if the participant had 16 years or more of education, 9 points or less if the participant had 8 to 15 years of education, and 6 points or less if the participant had 0 to 7 years of education; and (2) a CDR score of 0.5, which is comparable with the criteria used in the Japanese Alzheimer’s Disease Neuroimaging Initiative (J-ADNI) [20]. For the CN participants who were recruited from community-dwelling older adults and had not received a clinical diagnosis by a medical doctor specializing in dementia, the following criteria were additionally applied: (1) an MMSE score of 28 points or higher and (2) living independently. The exclusion criteria for MCI and CN were as follows: (1) the consumption of meals or any amino acid formulations, supplements, or beverages within 10 h before blood collection; (2) less than 6 years of education; (3) currently or previously treated for alcohol addiction; (4) cancer or liver cirrhosis; (5) on dialysis; (6) other neurodegenerative or mental disorders; and (7) assessment as ineligible by medical doctors. Figure 1 shows the summary of the study design.

### 2.4. PFAA Analysis

After overnight fasting, blood samples (5 mL) were collected from antecubital veins into tubes containing ethylenediaminetetraacetic acid disodium salt as an anticoagulant and were immediately (<1 min) placed in ice water or an ice-cold cooling container (Forte Grow Medical Co., Ltd., Tochigi, Japan). Plasma amino acid concentrations were measured by high-performance liquid chromatography (HPLC)–electrospray ionization (ESI)–mass spectrometry (MS) by precolumn derivatization. The analytical methods have been described elsewhere [17,18]. Concentrations of the following 19 amino acids were measured and analyzed: alanine (Ala), arginine (Arg), asparagine (Asn), citrulline (Cit), glutamine (Gln), glycine (Gly), histidine (His), isoleucine (Ile), leucine (Leu), lysine (Lys), methionine (Met), ornithine (Orn), phenylalanine (Phe), proline (Pro), serine (Ser), threonine (Thr), tryptophan (Trp), tyrosine (Tyr), and valine (Val).

### 2.5. Blood Biochemistry and APOE Genotyping

The height and weight of all participants were measured, and the body mass index (BMI) was calculated. Blood was collected under fasting conditions in the morning, and the following blood variables were measured in all study participants: albumin (Alb), fasting blood glucose (FBG), and creatinine. In addition, the following blood variables were measured in all MCI and CN participants: white blood cell (WBC) count, red blood cell (RBC) count, hemoglobin (Hb), hematocrit (Ht), platelet count, total protein (TP), prealbumin (TTR), C-reactive protein (CRP), aspartate aminotransferase (AST), alanine aminotransferase (ALT), γ-glutamyl transpeptidase (γ-GTP), urea nitrogen, uric acid, calcium, iron, folate, total cholesterol, LDL cholesterol, HDL cholesterol, triglyceride, insulin, and glycated hemoglobin (HbA1c).

Genomic DNA samples were extracted from peripheral blood leukocytes of all MCI participants using an automated DNA isolation system (QuickGene-Auto240L, Kurabo, Osaka, Japan). *APOE* genotypes (rs429358 and rs7412) were determined using TaqMan^®^ PCR Assays (Applied Biosystems, Foster City, CA, USA). According to previous studies [21,22], we defined participants with ε4 allele as the *APOE*-positive group and those without ε4 allele as the *APOE*-negative group.

### 2.6. Dataset Preparation

To prepare a training dataset, 120 MCI participants were selected according to the order in which blood samples were collected, and 120 of the 220 CN individuals were selected using propensity score matching based on sex and age. The remaining 99 MCI and 100 CN individuals were included in the validation dataset [23].

### 2.7. Statistical Analysis

#### 2.7.1. Characteristics and PFAA Profiles

The means and standard deviations or proportions were used to describe the distributions of characteristics and PFAA profiles for both individuals with MCI and CN controls. The Mann–Whitney U-test or Fisher’s exact test was used to assess significant differences in values between the MCI and CN groups.

#### 2.7.2. Receiver Operating Characteristic (ROC) Curve Analysis

ROC curve analysis was performed to determine the capabilities of uni- and multivariate models to discriminate between MCI and CN. The 95% confidence intervals (95% CI) of the AUC of ROC for the identification of MCI based on amino acid concentrations and ratios were also estimated using the empirical (nonparametric) method [24].

#### 2.7.3. PFAA Index Model Development

To construct the model, we used the concentrations of albumin and 19 PFAAs as the explanatory variables in the training dataset. Multivariate logistic regression analysis was performed to estimate the discriminatory ability of the model with regard to separating MCI from CN individuals. The maximum number of explanatory variables was restricted to less than seven to avoid potential multicollinearity. The PFAA index was defined as a linear predictor of the multivariate model using albumin and PFAA concentrations as variables. There were no missing values in the explanatory variables or the objective variable in the training dataset.

We confirmed that the variance inflation factor (VIF), which is the maximum of the diagonal element of the inverse matrix of the correlation coefficient matrix, did not choose inappropriate models with multicollinearity. We used a VIF of 10 as a cutoff value to distinguish between “high” and “low” based on a previous study [25].

#### 2.7.4. Model Selection

For model selection, bootstrapping was used to quantify any optimism in the predictive performance of the developed model and what performance might be expected in other participants from the underlying source population from which the development sample originated [23]. The bootstrapping is summarized in the following 4 steps [26]:

1 Generate a bootstrap sample by sampling 120 paired participants from the original sample (training dataset).
2 Develop a model using the bootstrap sample:
  a Determine the AUC of ROC of this model on the bootstrap sample (bootstrap performance).
  b Determine the AUC of ROC of the bootstrap model in the original sample (test performance).
3 Calculate the optimism as the difference between the bootstrap performance and the test performance.
  Repeat steps 1–3 100 times. Average the estimates of optimism.
4 The optimism-corrected performance was calculated by subtracting the optimism from the apparent performance. We selected models with the optimism-corrected performance (AUC of ROC) in the top 50 for validation.

#### 2.7.5. Model Validation

The selected models’ performances were validated in the independent validation dataset. There were no missing values in the explanatory variables or objective variables in the validation dataset.

For the correlation analysis, we used Pearson’s r (denoted as r) or Spearman’s roh (denoted as rs) according to the distribution of the variable.

### 2.8. Software

All statistical and multivariate analyses were performed within the R (ver. 3.5.3) platform (R Foundation for Statistical Computing, Vienna, Austria) and GraphPad Prism V8.4.3 (GraphPad Software, Inc., San Diego, CA, USA) statistical software.

## 3. Results

### 3.1. Characteristics and PFAA Profiles of MCI and CN Participants

Table 1 summarizes the characteristics of the MCI and CN individuals (MCI, *n* = 219; CN, *n* = 220). Table 2 shows the concentrations of albumin and 19 PFAAs in the training and validation datasets. Almost all of the amino acid concentrations measured in this study were within the previously reported reference intervals. In the training dataset, significantly lower (*p* < 0.05) concentrations of albumin and three amino acids (His, Met, and Lys) were found in the MCI group than in the CN group (Table 2). Additionally, in the combined training and validation dataset, the concentrations of albumin and eight amino acids (Asn, His, Thr, Val, Met, Lys, Leu, and Phe) were significantly lower (*p* < 0.05) in the MCI group than in the CN group (Table 2).

**Figure 2:**
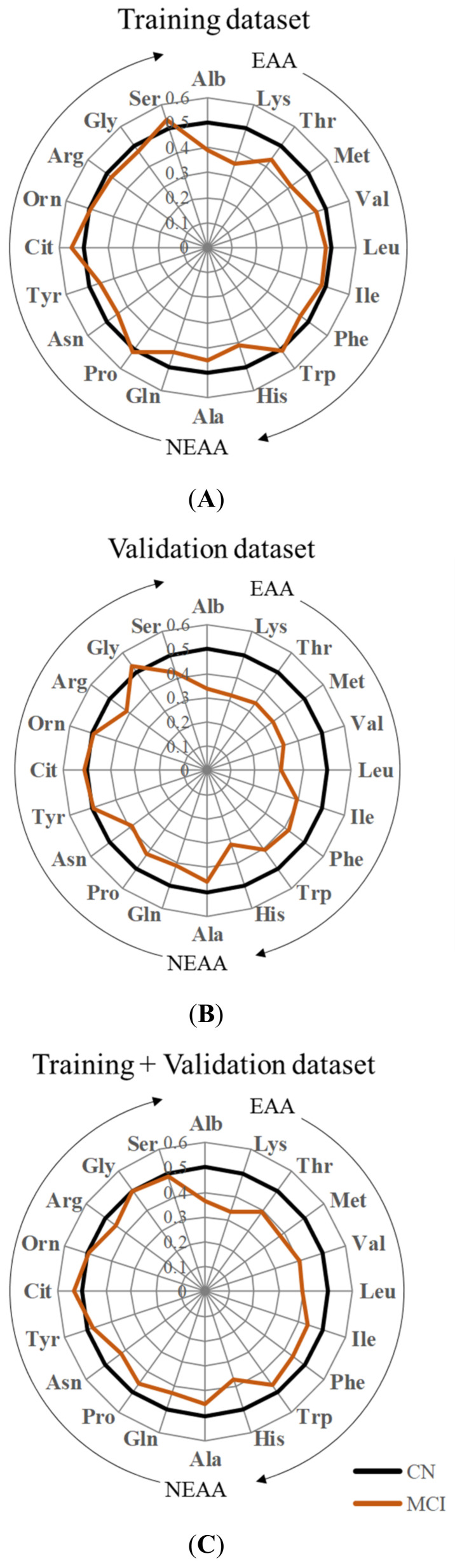
Albumin and PFAA profile of MCI and CN groups. (**A**) Training dataset (120 MCI and 120 CN); (**B**) validation dataset (99 MCI and 100 CN), and (**C**) training + validation dataset (219 MCI and 220 CN). The results of receiver operating characteristic (ROC) curve analysis of albumin and plasma free amino acids (PFAAs) in the training dataset (120 MCI and matching 120 CN) (**A**), the validation dataset (99 MCI and remaining 100 CN) (**B**), and the training and validation dataset (219 MCI and 220 CN) (**C**). Axes show the area under the curve (AUC) of the ROC for albumin and each amino acid for the discrimination of MCI from CN. Black bold lines indicate the point at which the AUC of ROC = 0.5. The MCI labels were fixed as positive class labels. Therefore, an AUC of ROC value < 0.5 indicated the level was lower in the MCI group, whereas a value > 0.5 indicated that the level was higher in the MCI group. EAA; essential amino acids, NEAA; nonessential amino acids.

Additionally, we applied ROC curve analysis to albumin and 19 PFAAs in the training and/or validation datasets (Figure 2). The MCI labels were fixed as positive class labels. Therefore, an area under the ROC curve (AUC of ROC) value <0.5 indicated that the amino acid level was lower in the MCI group than in the CN group, whereas an AUC of ROC value >0.5 indicated the reverse. We confirmed that the concentrations of albumin and some essential amino acid concentrations, such as Lys and His, were lower in the MCI group than in the CN group in both datasets.

### 3.2. PFAA Index Development

To discriminate MCI participants from CN individuals, we calculated the optimal PFAA indices by multiple logistic regression analysis in the training dataset. The top 50 models were selected based on the optimism-corrected AUC of ROC. The optimism-corrected AUC of ROC values were nearly identical (0.72–0.70) in the top 50 models. We validated the performance of the top 50 models in the validation dataset and selected the model composed of Alb, Ser, Thr, Cit, Lys, and Trp, hereinafter referred to as the “PFAA index”, as a representative model. In addition, we confirmed that the VIFs were lower than 10 in all the top 50 models. The highest VIF was of the representative model; this VIF of 1.73 suggested that no multicollinearity existed in the model.

Furthermore, to estimate the effects of potential confounding factors, logistic regression analyses adding age and/or sex and/or BMI and/or MMSE score to the PFAA index were performed (Table 3). No obvious change in significance was observed when those factors were added into the model, suggesting that the plasma amino acid and albumin concentrations observed in the MCI group were independent of age, sex, BMI, and MMSE score.

### 3.3. Discriminatory Performance of the PFAA Index

**Figure 3:**
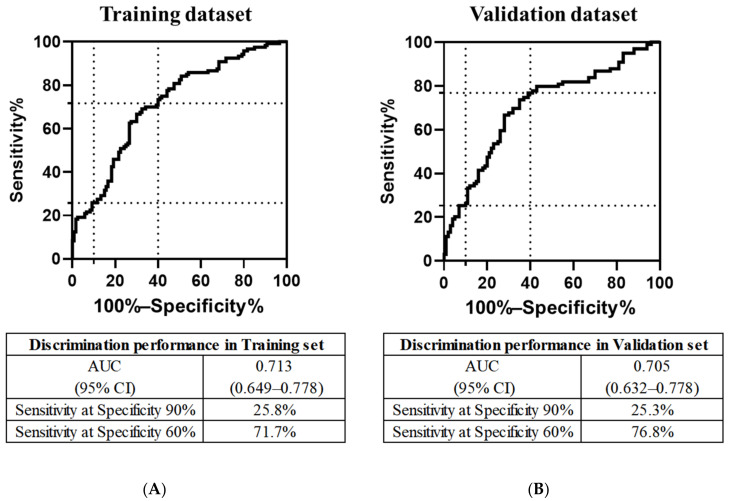
ROC curves of the PFAA index ^1^. (**A**) ROC curves of the PFAA index for MCI participants compared with CN participants in the training dataset (120 MCI and matching 120 CN) and (**B**) the validation dataset (99 MCI and 100 remaining CN). The vertical dotted lines show specificities of 90% and 60%. The horizontal dotted lines show the sensitivities at specificities of 90% and 60%. ^1^ The PFAA index consists of the following variables: Alb, Ser, Thr, Cit, Lys, and Trp.

To evaluate the performance of the PFAA index with regard to discriminating MCI from CN individuals, ROC curves in the training and validation datasets were constructed (Figure 3). In the training dataset, the AUC of the PFAA index for detecting MCI was 0.713 (95% CI, 0.648–0.778). The sensitivities of the PFAA index at 90% and 60% specificities were 25.8% and 71.7%, respectively (Figure 3A). In the validation dataset, the AUC of the PFAA index was 0.705 (95% CI, 0.632–0.778). The sensitivities of the PFAA index at specificities of 90% and 60% were 25.3% and 76.8%, respectively (Figure 3B). The performance metrics of other indices consisting of albumin and other PFAAs and other indices consisting of only PFAAs are shown in Supplementary Table S1.

### 3.4. Association of the PFAA Index with MCI Risk Factors and Other Variables

We evaluated the correlations between the PFAA index values and BMI in MCI and CN individuals. In the MCI group, there was no significant correlation between the PFAA index and BMI (r = −0.071, *p* = 0.316) (Supplementary Figure S1A). In the CN group, we detected a statistically significant but very weak correlation between the PFAA index and BMI (r = −0.168, *p* = 0.0122). Furthermore, we evaluated the relationship between the PFAA index values and the *APOE* genotypes in the MCI group and found no significant differences in the PFAA index values between the *APOE*-positive (with the ε4 allele) and *APOE*-negative (without the ε4 allele) groups (Supplementary Figure S1B). As an exploratory analysis, the relationship between the PFAA index and other MCI risk factors, such as diabetes mellitus, hypertension, low-density lipoprotein cholesterol level, and triglyceride level, were analyzed, and no associations were found (Supplementary Figure S2).

## 4. Discussion

In this multicenter clinical study, we generated an index with PFAA and albumin concentrations as variables to identify individuals who are at high risk for MCI in the population over the age of 50 years. Although the PFAA index may serve as a screening tool, the sensitivity and specificity observed in the present study may be suboptimal and leave room for further research. The PFAA index was independent of confounding factors such as age, sex, BMI, and MMSE score. No significant differences in the PFAA index values between the *APOE*-positive and *APOE*-negative status were found. To date, several researchers have reported the relationship between changes in the PFAA profile and dementia [27,28,29]; however, the results have been inconsistent. One recent metabolomics study using NMR based on eight prospective cohorts with 22,623 participants demonstrated that lower levels of serum branched-chain amino acids (BCAAs) such as valine were associated with an increased risk of both all types of dementia and AD [29]. While this result may provide some indication of the relationship between protein nutrition and the risk of developing dementia and AD in the future, our study did not find significantly lower levels of BCAAs in the MCI group. This discrepancy may be due to the difference in research design between longitudinal and cross-sectional studies. Our study did not target the onset of dementia but instead explored the differences between MCI and CN individuals in a cross-sectional study. Since our study will continue to follow up on the onset of AD for several years, we would like to observe plasma BCAAs in the future analysis. Moreover, in comparison with other studies, sample collection and measurement methods may have also had an effect. Metabolites, including amino acids, are unstable in ambient blood samples due to enzymatic activity [30]. The degradation pattern of metabolites also differs depending on whether the sample is serum or plasma. Therefore, our index, which used plasma samples collected under strict sample management, might have detected differences that previous serum-based studies did not detect.

It is important to note that the concentrations of albumin and some essential amino acids, such as Lys, His, and Thr, were lower in the MCI group. It is known that PFAA profiles change due to various factors, such as lifestyle-related diseases [31], visceral fat accumulation [32], and a decrease in muscle skeletal mass [33]. Additionally, since amino acids are the building blocks of proteins, insufficient protein intake could lead to low concentrations of PFAAs, especially essential amino acids [34,35]. A recent animal experiment demonstrated that a low-protein diet resulted in decreased concentrations of plasma essential amino acids, and led to cognitive dysfunction in old mice, such as learning disabilities, disinhibition, and hyperactive behavior, along with loss of neurotransmitters in the brain [36]. Notably, in a mice model of neurodegenerative tauopathies, the low-protein diet resulted in the down-regulated expression of synaptic components and a modest acceleration of brain atrophy, while administration of essential amino acids reversed these conditions [37].

The protein intake level in some elderly individuals is insufficient [38,39,40,41]. Issues such as decreased appetite [42,43], dysphagia [44], reduced strength in the muscles needed for meat consumption [45,46], and periodontal disease [47] have been noted as causes. According to a study in the Netherlands, approximately 14% of community-dwelling elderly adults with newly diagnosed dementia were at risk of malnutrition [48]. Those authors pointed out that assessment of nutritional status should be included in the comprehensive assessment of AD patients. Additionally, in past studies, those with cognitive decline after 70 years of age had lower body weight, insufficient diet, and lower physical activity levels at midlife than those without cognitive decline [49]. Recently, clinical evidence of the relationship between intake of individual specific amino acids and cognitive function has been emerging [50,51]. Considering that there are various factors that lead to MCI, such as diet, exercise, vascular risk, education, stress, and genetic background, the current PFAA index may be useful in identifying people with MCI due to dietary protein insufficiency. It will be important in the future to investigate the relationships between dietary intake, PFAA profiles, and cognitive status in these populations.

This study has several limitations. First, the number of participants in this study was not large enough to enable a stratified analysis. In addition, we could not stratify MCI participants based on the underlying molecular pathology because our study did not include molecular imaging or CSF biomarkers. Additional studies in the future with these data may provide further insight into possible differences in PFAA profiles between different pathological backgrounds. Secondly, this study did not obtain data on additional factors that may affect PFAAs and cognitive function, such as dietary surveys, measurements of muscle mass, and physical activity. It will be important to examine the correlation of these data in future studies, especially with protein nutritional status. Thirdly, due to the cross-sectional study design, the current study could not investigate the relationship between the PFAA index and the onset of dementia. Follow-up of the participants is currently ongoing, and the relationship with the conversion to AD will be analyzed in the future.

## 5. Conclusions

This multicenter clinical study underscores the importance of clinically evaluating PFAA and albumin profiles to assess the MCI risk in a general population over the age of 50. We generated an index with PFAA and albumin concentrations as variables to identify individuals who are at high risk for MCI. The PFAA index was independent of confounding factors such as age, sex, BMI, and MMSE score. Additionally, no significant differences in the PFAA index values between the *APOE*-positive and *APOE*-negative status were observed. Further studies are needed to clarify the relationship between the PFAA index, nutritional intake, and cognitive changes.

## 6. Patents

T.I., Y.Y. (Yuki Yano) and W.S. filed a patent related to this work, filed by Niigata University and Ajinomoto Co. Inc., Kanagawa, Japan (application no. WO2020/067386, published on 2 April 2020).

## Figures and Tables

**Table 1:** Characteristics of MCI and CN individuals.

		Training Dataset			Validation Dataset		
		MCI (N = 120)	CN (N = 120)	*p*-Value ^1^	MCI (N = 99)	CN (N = 100)	*p*-Value ^1^
Sex				0.683			<0.001
Male	N (%)	39 (32.5)	43 (35.8)		35 (35.4)	60 (60.0)	
Female	N (%)	81 (67.5)	77 (64.2)		64 (64.6)	40 (40.0)	
Age, years	Mean ± SD	80.3 ± 5.5	79.3 ± 5.4	0.159	78.6 ± 5.8	72.8 ± 6.2	<0.001
	(range)	(67–96)	(64–91)		(63–89)	(51–80)	
BMI, kg/m^2^	Mean ± SD	22.6 ± 3.8 ^2^	22.6 ± 2.9	0.717	22.6 ± 3.4 ^2^	23.1 ± 2.9	0.403
MMSE	Mean ± SD	26.9 ± 2.0	29.3 ± 0.8	<0.001	26.7 ± 2.1	29.4 ± 0.7	<0.001
GDS-15	Mean ± SD	1.5 ± 1.4	1.7 ± 1.6	0.624	1.8 ± 1.4	1.7 ± 1.7	0.280
Educational background, years	Mean ± SD	11.2 ± 2.4	12.7 ± 2.4 ^2^	<0.001	11.9 ± 2.5	13.2 ± 2.7 ^2^	0.004
*APOE* genotype							
positive (with ε4 allele)	N (%)	42 (35)	-		33 (33.3)	-	
negative (without ε4 allele)	N (%)	78 (65)	-		60 (60.6)	-	
missing	N (%)	0 (0)	-		6 (6.1)	-	

^1^ The sex distribution was compared between the MCI and CN groups with Fisher’s exact test. For the other variables, the differences between the MCI and CN groups were tested by the Mann–Whitney U-test. ^2^ For BMI, 11 and 8 data points were missing in the MCI group in the training dataset and the validation dataset, respectively. For educational background, 55 and 37 data points were missing in the CN group in the training dataset and in the validation dataset, respectively.

**Table 2:** Albumin (g/dL) and PFAA concentrations (μmol/L) in the MCI and CN groups.

	Training Set			Validation Set			Training + Validation Set		
	MCI (N = 120)	CN (N = 120)		MCI (N = 99)	CN (N = 100)		MCI (N = 219)	CN (N = 220)	
	Mean ± SD	Mean ± SD	*p*-Value ^1^	Mean ± SD	Mean ± SD	*p*-Value ^1^	Mean ± SD	Mean ± SD	*p*-Value ^1^
Alb	4.2 ± 0.3	4.3 ± 0.3	0.003	4.2 ± 0.3	4.4 ± 0.3	<0.001	4.2 ± 0.3	4.4 ± 0.3	<0.001
Lys	178.4 ± 29.8	192.2 ± 28.4	<0.001	182.1 ± 29.4	200.2 ± 32.6	<0.001	180.1 ± 29.6	195.8 ± 30.6	<0.001
Thr	110.1 ± 22.4	115.2 ± 22.2	0.080	111.3 ± 24.6	124.2 ± 26.7	<0.001	110.7 ± 23.4	119.3 ± 24.7	<0.001
Met	23.7 ± 4.9	24.7 ± 4.2	0.024	23.7 ± 5.4	26.1 ± 4.8	<0.001	23.7 ± 5.1	25.3 ± 4.5	<0.001
Val	204.8 ± 42.5	207.9 ± 32.5	0.284	202.1 ± 43.0	225.3 ± 42.2	<0.001	203.6 ± 42.7	215.8 ± 38.1	<0.001
Leu	107.5 ± 24.0	109.0 ± 20.9	0.540	107.0 ± 26.9	122.6 ± 24.9	<0.001	107.3 ± 25.3	115.2 ± 23.7	<0.001
Ile	57.8 ± 14.7	57.9 ± 12.8	0.670	57.9 ± 15.8	63.0 ± 15.5	0.007	57.8 ± 15.2	60.2 ± 14.3	0.030
Phe	61.6 ± 12.4	62.0 ± 8.8	0.334	59.2 ± 10.4	61.5 ± 9.2	0.045	60.5 ± 11.6	61.8 ± 9.0	0.040
Trp	50.2 ± 9.9	49.9 ± 8.4	0.765	49.6 ± 8.9	52.0 ± 7.9	0.023	49.9 ± 9.4	50.9 ± 8.2	0.177
His	76.7 ± 9.2	78.9 ± 8.7	0.018	76.1 ± 10.4	82.1 ± 9.5	<0.001	76.4 ± 9.7	80.4 ± 9.2	<0.001
Ala	345.2 ± 90.4	356.0 ± 77.6	0.161	351.2 ± 90.2	359.1 ± 77.9	0.328	348.0 ± 90.2	357.4 ± 77.5	0.093
Gln	595.8 ± 71.4	606.4 ± 58.0	0.107	596.1 ± 67.9	613.3 ± 54.0	0.032	595.9 ± 69.7	609.5 ± 56.2	0.009
Pro	144.1 ± 56.1	142.1 ± 43.5	0.651	141.9 ± 50.3	149.8 ± 45.1	0.072	143.1 ± 53.4	145.6 ± 44.3	0.117
Asn	45.1 ± 7.6	46.6 ± 7.8	0.139	44.9 ± 6.9	47.5 ± 7.5	0.007	45.0 ± 7.3	47.0 ± 7.6	0.004
Tyr	63.7 ± 14.3	64.1 ± 11.0	0.221	64.0 ± 12.9	64.1 ± 12.8	0.989	63.9 ± 13.6	64.1 ± 11.8	0.400
Cit	39.5 ± 10.9	37.6 ± 10.1	0.185	37.5 ± 10.9	36.4 ± 8.9	0.774	38.6 ± 10.9	37.1 ± 9.6	0.200
Orn	58.6 ± 15.8	58.9 ± 18.5	0.936	57.5 ± 13.6	58.0 ± 13.3	0.915	58.1 ± 14.8	58.5 ± 16.4	0.970
Arg	93.6 ± 18.8	94.6 ± 18.7	0.569	92.4 ± 18.2	97.3 ± 19.7	0.044	93.1 ± 18.5	95.8 ± 19.1	0.079
Gly	221.7 ± 59.1	223.7 ± 52.8	0.548	230.9 ± 60.2	225.3 ± 62.2	0.452	225.8 ± 59.6	224.4 ± 57.1	0.974
Ser	108.5 ± 18.6	105.7 ± 18.8	0.297	104.5 ± 20.3	110.3 ± 21.3	0.058	106.7 ± 19.4	107.8 ± 20.1	0.629

^1^ The *p*-values were obtained by performing a Mann–Whitney U-test between the MCI and CN groups. There was no missing albumin or PFAA values in the training dataset or the validation dataset.

**Table 3:** Independence between the PFAA index and potential confounders.

	*p*-Value for a Variable in Logistic Regression															
Variable	Base Model	+Age	+Sex	+MMSE	+BMI	+A, S	+A, M	+A, B	+S, M	+S, B	+M, B	+A, S, M	+A, S, B	+A, M, B	+S, M, B	+A, S, M, B
PFAA index	**3.8 × 10^−8^**	**7.6 × 10^−8^**	**3.8 × 10^−8^**	**3.3 × 10^−5^**	**4.4 × 10^−8^**	**7.9 × 10^−8^**	**2.1 × 10^−5^**	**1.2 × 10^−7^**	**3.4 × 10^−5^**	**4.7 × 10^−8^**	**4.2 × 10^−5^**	**2.2 × 10^−5^**	**1.4 × 10^−7^**	**2.8 × 10^−5^**	**4.3 × 10^−5^**	**2.9 × 10^−5^**
Age		0.84				0.79	0.19	0.65				0.21	0.60	0.24		0.26
Sex			0.57			0.56			0.60	0.50		0.68	0.47		0.53	0.60
MMSE				**3.6 × 10^−12^**			**6.0 × 10^−12^**		**3.9 × 10^−12^**		**2.0 × 10^−11^**	**6.4 × 10^−12^**		**3.0 × 10^−11^**	**2.1 × 10^−11^**	**3.1 × 10^−11^**
BMI					0.46			0.49		0.46	0.85		0.49	0.79	0.86	0.79

Bold text indicates statistical significance with a *p*-value less than 0.05. A, age; S, sex; M, MMSE; B, BMI.

## Data Availability

The data presented in this study are available on request from the corresponding author. The data are not publicly available due to privacy and ethical reasons.

## Supplementary Materials

The following are available online at https://www.mdpi.com/article/10.3390/nu14030637/s1, Table S1: Performance of models with different variables, Figure S1: Relationships of the PFAA index with BMI and *APOE*, Figure S2: Relationships between PFAA index and lifestyle-related diseases.

## References

[1] Sperling R.A., Aisen P.S., Beckett L.A., Bennett D.A., Craft S., Fagan A.M., Iwatsubo T., Jack C.R., Kaye J., Montine T.J. (2011). Toward defining the preclinical stages of Alzheimer’s disease: Recommendations from the National Institute on Aging-Alzheimer’s Association workgroups on diagnostic guidelines for Alzheimer’s disease. Alzheimers Dement..

[2] El-Hayek Y.H., Wiley R.E., Khoury C.P., Daya R.P., Ballard C., Evans A.R., Karran M., Molinuevo J.L., Norton M., Atri A. (2019). Tip of the Iceberg: Assessing the Global Socioeconomic Costs of Alzheimer’s Disease and Related Dementias and Strategic Implications for Stakeholders. J. Alzheimers Dis..

[3] Leroi I., Watanabe K., Hird N., Sugihara T. (2018). “Psychogeritechnology” in Japan: Exemplars from a super-aged society. Int. J. Geriatr. Psychiatry.

[4] Livingston G., Sommerlad A., Orgeta V., Costafreda S.G., Huntley J., Ames D., Ballard C., Banerjee S., Burns A., Cohen-Mansfield J. (2017). Dementia prevention, intervention, and care. Lancet.

[5] Ngandu T., Lehtisalo J., Solomon A., Levalahti E., Ahtiluoto S., Antikainen R., Backman L., Hanninen T., Jula A., Laatikainen T. (2015). A 2 year multidomain intervention of diet, exercise, cognitive training, and vascular risk monitoring versus control to prevent cognitive decline in at-risk elderly people (FINGER): A randomised controlled trial. Lancet.

[6] Li J., Joshi P., Ang T.F.A., Liu C., Auerbach S., Devine S., Au R. (2021). Mid- to Late-Life Body Mass Index and Dementia Risk: 38 Years of Follow-up of the Framingham Study. Am. J. Epidemiol..

[7] Wei K., Nyunt M.S., Gao Q., Wee S.L., Yap K.B., Ng T.P. (2018). Association of Frailty and Malnutrition With Long-term Functional and Mortality Outcomes Among Community-Dwelling Older Adults: Results From the Singapore Longitudinal Aging Study 1. JAMA Netw. Open.

[8] Nes M., Sem S.W., Rousseau B., Bjorneboe G.E., Engedal K., Trygg K., Pedersen J.I. (1988). Dietary intakes and nutritional status of old people with dementia living at home in Oslo. Eur. J. Clin. Nutr..

[9] Sanders C.L., Wengreen H.J., Schwartz S., Behrens S.J., Corcoran C., Lyketsos C.G., Tschanz J.T., Cache County I. (2018). Nutritional Status is Associated with Severe Dementia and Mortality: The Cache County Dementia Progression Study. Alzheimer Dis. Assoc. Disord..

[10] Thomas D.E., Chung A.O.K.O., Dickerson J.W., Tidmarsh S.F., Shaw D.M. (1986). Tryptophan and nutritional status of patients with senile dementia. Psychol. Med..

[11] Goodwin J.S., Goodwin J.M., Garry P.J. (1983). Association between nutritional status and cognitive functioning in a healthy elderly population. JAMA.

[12] La Rue A., Koehler K.M., Wayne S.J., Chiulli S.J., Haaland K.Y., Garry P.J. (1997). Nutritional status and cognitive functioning in a normally aging sample: A 6-y reassessment. Am. J. Clin. Nutr..

[13] Roberts R.O., Roberts L.A., Geda Y.E., Cha R.H., Pankratz V.S., O’Connor H.M., Knopman D.S., Petersen R.C. (2012). Relative intake of macronutrients impacts risk of mild cognitive impairment or dementia. J. Alzheimers Dis..

[14] Fernando W., Rainey-Smith S.R., Gardener S.L., Villemagne V.L., Burnham S.C., Macaulay S.L., Brown B.M., Gupta V.B., Sohrabi H.R., Weinborn M. (2018). Associations of Dietary Protein and Fiber Intake with Brain and Blood Amyloid-beta. J. Alzheimers Dis..

[15] Licher S., Ahmad S., Karamujic-Comic H., Voortman T., Leening M.J.G., Ikram M.A., Ikram M.K. (2019). Genetic predisposition, modifiable-risk-factor profile and long-term dementia risk in the general population. Nat. Med..

[16] Keller U. (2019). Nutritional Laboratory Markers in Malnutrition. J. Clin. Med..

[17] Nakayama A., Imaizumi A., Yoshida H. (2019). Methods for Absolute Quantification of Human Plasma Free Amino Acids by High-Performance Liquid Chromatography/Electrospray Ionization Mass Spectrometry Using Precolumn Derivatization. Methods Mol. Biol..

[18] Yoshida H., Kondo K., Yamamoto H., Kageyama N., Ozawa S., Shimbo K., Muramatsu T., Imaizumi A., Mizukoshi T., Masuda J. (2015). Validation of an analytical method for human plasma free amino acids by high-performance liquid chromatography ionization mass spectrometry using automated precolumn derivatization. J. Chromatogr. B Analyt. Technol. Biomed. Life Sci..

[19] Yamamoto H., Kondo K., Tanaka T., Muramatsu T., Yoshida H., Imaizumi A., Nagao K., Noguchi Y., Miyano H. (2016). Reference intervals for plasma-free amino acid in a Japanese population. Ann. Clin. Biochem..

[20] Iwatsubo T., Iwata A., Suzuki K., Ihara R., Arai H., Ishii K., Senda M., Ito K., Ikeuchi T., Kuwano R. (2018). Japanese and North American Alzheimer’s Disease Neuroimaging Initiative studies: Harmonization for international trials. Alzheimers Dement..

[21] Lopez O.L., Jagust W.J., Dulberg C., Becker J.T., DeKosky S.T., Fitzpatrick A., Breitner J., Lyketsos C., Jones B., Kawas C. (2003). Risk factors for mild cognitive impairment in the Cardiovascular Health Study Cognition Study: Part 2. Arch. Neurol..

[22] Small G.W., Ercoli L.M., Silverman D.H., Huang S.C., Komo S., Bookheimer S.Y., Lavretsky H., Miller K., Siddarth P., Rasgon N.L. (2000). Cerebral metabolic and cognitive decline in persons at genetic risk for Alzheimer’s disease. Proc. Natl. Acad. Sci. USA.

[23] Moons K.G., Altman D.G., Reitsma J.B., Ioannidis J.P., Macaskill P., Steyerberg E.W., Vickers A.J., Ransohoff D.F., Collins G.S. (2015). Transparent Reporting of a multivariable prediction model for Individual Prognosis or Diagnosis (TRIPOD): Explanation and elaboration. Ann. Intern. Med..

[24] DeLong E.R., DeLong D.M., Clarke-Pearson D.L. (1988). Comparing the areas under two or more correlated receiver operating characteristic curves: A nonparametric approach. Biometrics.

[25] Craney T.A., Surles J.G. (2002). Model-Dependent Variance Inflation Factor Cutoff Values. Qual. Eng..

[26] Harrell F.E., Lee K.L., Mark D.B. (1996). Multivariable prognostic models: Issues in developing models, evaluating assumptions and adequacy, and measuring and reducing errors. Stat. Med..

[27] Van der Lee S.J., Teunissen C.E., Pool R., Shipley M.J., Teumer A., Chouraki V., Melo van Lent D., Tynkkynen J., Fischer K., Hernesniemi J. (2018). Circulating metabolites and general cognitive ability and dementia: Evidence from 11 cohort studies. Alzheimers Dement..

[28] Ravaglia G., Forti P., Maioli F., Bianchi G., Martelli M., Talerico T., Servadei L., Zoli M., Mariani E. (2004). Plasma amino acid concentrations in patients with amnestic mild cognitive impairment or Alzheimer disease. Am. J. Clin. Nutr..

[29] Tynkkynen J., Chouraki V., van der Lee S.J., Hernesniemi J., Yang Q., Li S., Beiser A., Larson M.G., Saaksjarvi K., Shipley M.J. (2018). Association of branched-chain amino acids and other circulating metabolites with risk of incident dementia and Alzheimer’s disease: A prospective study in eight cohorts. Alzheimers Dement..

[30] Takehana S., Yoshida H., Ozawa S., Yamazaki J., Shimbo K., Nakayama A., Mizukoshi T., Miyano H. (2016). The effects of pre-analysis sample handling on human plasma amino acid concentrations. Clin. Chim. Acta.

[31] Yamakado M., Nagao K., Imaizumi A., Tani M., Toda A., Tanaka T., Jinzu H., Miyano H., Yamamoto H., Daimon T. (2015). Plasma Free Amino Acid Profiles Predict Four-Year Risk of Developing Diabetes, Metabolic Syndrome, Dyslipidemia, and Hypertension in Japanese Population. Sci. Rep..

[32] Yamakado M., Tanaka T., Nagao K., Ishizaka Y., Mitushima T., Tani M., Toda A., Toda E., Okada M., Miyano H. (2012). Plasma amino acid profile is associated with visceral fat accumulation in obese Japanese subjects. Clin. Obes..

[33] Yamada M., Kimura Y., Ishiyama D., Nishio N., Tanaka T., Ohji S., Otobe Y., Koyama S., Sato A., Suzuki M. (2018). Plasma Amino Acid Concentrations Are Associated with Muscle Function in Older Japanese Women. J. Nutr. Health Aging.

[34] Fujita Y., Yamamoto T., Rikimaru T., Inoue G. (1979). Effect of low protein diets on free amino acids in plasma of young men: Effect of wheat gluten diet. J. Nutr. Sci. Vitam..

[35] Fujita Y., Yoshimura Y., Inoue G. (1978). Effect of low-protein diets on free amino acids in plasma of young men: Effect of protein quality with maintenance or excess energy intake. J. Nutr. Sci. Vitam..

[36] Sato H., Tsukamoto-Yasui M., Takado Y., Kawasaki N., Matsunaga K., Ueno S., Kanda M., Nishimura M., Karakawa S., Isokawa M. (2020). Protein Deficiency-Induced Behavioral Abnormalities and Neurotransmitter Loss in Aged Mice Are Ameliorated by Essential Amino Acids. Front. Nutr..

[37] Sato H., Takado Y., Toyoda S., Tsukamoto-Yasui M., Minatohara K., Takuwa H., Urushihata T., Takahashi M., Shimojo M., Ono M. (2021). Neurodegenerative processes accelerated by protein malnutrition and decelerated by essential amino acids in a tauopathy mouse model. Sci. Adv..

[38] Bauer J., Biolo G., Cederholm T., Cesari M., Cruz-Jentoft A.J., Morley J.E., Phillips S., Sieber C., Stehle P., Teta D. (2013). Evidence-based recommendations for optimal dietary protein intake in older people: A position paper from the PROT-AGE Study Group. J. Am. Med. Dir. Assoc..

[39] Deutz N.E., Bauer J.M., Barazzoni R., Biolo G., Boirie Y., Bosy-Westphal A., Cederholm T., Cruz-Jentoft A., Krznaric Z., Nair K.S. (2014). Protein intake and exercise for optimal muscle function with aging: Recommendations from the ESPEN Expert Group. Clin. Nutr..

[40] Paddon-Jones D., Leidy H. (2014). Dietary protein and muscle in older persons. Curr. Opin. Clin. Nutr. Metab. Care.

[41] Volpi E., Campbell W.W., Dwyer J.T., Johnson M.A., Jensen G.L., Morley J.E., Wolfe R.R. (2013). Is the optimal level of protein intake for older adults greater than the recommended dietary allowance?. J. Gerontol. A Biol. Sci. Med. Sci..

[42] Pelchat M.L., Schaefer S. (2000). Dietary monotony and food cravings in young and elderly adults. Physiol. Behav..

[43] Pilgrim A.L., Robinson S.M., Sayer A.A., Roberts H.C. (2015). An overview of appetite decline in older people. Nurs. Older People.

[44] Kawashima K., Motohashi Y., Fujishima I. (2004). Prevalence of dysphagia among community-dwelling elderly individuals as estimated using a questionnaire for dysphagia screening. Dysphagia.

[45] Maeda K., Akagi J. (2015). Decreased tongue pressure is associated with sarcopenia and sarcopenic dysphagia in the elderly. Dysphagia.

[46] Yamaguchi K., Tohara H., Hara K., Nakane A., Kajisa E., Yoshimi K., Minakuchi S. (2018). Relationship of aging, skeletal muscle mass, and tooth loss with masseter muscle thickness. BMC Geriatr..

[47] Polzer I., Schimmel M., Muller F., Biffar R. (2010). Edentulism as part of the general health problems of elderly adults. Int. Dent. J..

[48] Droogsma E., van Asselt D.Z., Scholzel-Dorenbos C.J., van Steijn J.H., van Walderveen P.E., van der Hooft C.S. (2013). Nutritional status of community-dwelling elderly with newly diagnosed Alzheimer’s disease: Prevalence of malnutrition and the relation of various factors to nutritional status. J. Nutr. Health Aging.

[49] Wagner M., Grodstein F., Proust-Lima C., Samieri C. (2020). Long-Term Trajectories of Body Weight, Diet, and Physical Activity From Midlife Through Late Life and Subsequent Cognitive Decline in Women. Am. J. Epidemiol..

[50] Kinoshita K., Otsuka R., Takada M., Tsukamoto-Yasui M., Nishita Y., Tange C., Tomida M., Shimokata H., Kuzuya M., Imaizumi A. (2021). The Association between Dietary Amino Acid Intake and Cognitive Decline 8 Years Later in Japanese Community-Dwelling Older Adults. J. Nutr. Health Aging.

[51] Suzuki H., Yamashiro D., Ogawa S., Kobayashi M., Cho D., Iizuka A., Tsukamoto-Yasui M., Takada M., Isokawa M., Nagao K. (2020). Intake of Seven Essential Amino Acids Improves Cognitive Function and Psychological and Social Function in Middle-Aged and Older Adults: A Double-Blind, Randomized, Placebo-Controlled Trial. Front. Nutr..

