# Supplementary material for: Development of a Novel Nutrition-Related Multivariate Biomarker for Mild Cognitive Impairment Based on the Plasma Free Amino Acid Profile
Source: Nutrients. 2022 Feb 1;14(3):637. doi: 10.3390/nu14030637 (PMC8840028; doi:10.3390/nu14030637)
Supplement: Supplementary file 1 [file nutrients-14-00637-s001.zip › nutrients-1550330-supplementary.pdf]

Supplementary materials for "Development of a Novel Nutrition-Related Multivariate Biomarker for Mild Cognitive Impairment Based on the Plasma Free Amino Acid Profile"

Table S1. Performance of models with different variables.

							Training dataset			Validation dataset		
							MCI (N=120) CN (N=120)			MCI (N=99) CN (N=100)		
**Variable type**	**PFAA index mode variables[1]**						**AUC of ROC**	**95%CI**	**p-value [2]**	**AUC of ROC**	**95%CI**	**p-value [2]**
Alb+PFAA	Alb	Ser	Thr	Cit	Lys	Trp	0.71	(0.65 - 0.78)	< 0.001	0.70	(0.63 - 0.78)	< 0.001
Alb+PFAA	Alb	Ser	His	Thr	Lys	Trp	0.71	(0.65 - 0.78)	< 0.001	0.70	(0.63 - 0.77)	< 0.001
Alb+PFAA	Alb	Ser	His	Cit	Lys	Trp	0.71	(0.65 - 0.78)	< 0.001	0.69	(0.62 - 0.77)	< 0.001
Alb+PFAA	Alb	Ser	Thr	Arg	Lys	Trp	0.72	(0.65 - 0.78)	< 0.001	0.69	(0.62 - 0.76)	< 0.001
Alb+PFAA	Alb	Ser	Cit	Met	Lys	Trp	0.72	(0.66 - 0.78)	< 0.001	0.68	(0.61 - 0.76)	< 0.001
PFAA only	Ser	Gln	Thr	Cit	Lys	Trp	0.70	(0.64 - 0.77)	< 0.001	0.66	(0.58 - 0.74)	< 0.001
PFAA only	Ser	Thr	Cit	Met	Lys	Trp	0.70	(0.64 - 0.77)	< 0.001	0.65	(0.58 - 0.73)	< 0.001
PFAA only	Ser	Thr	Cit	Orn	Lys	Trp	0.70	(0.63 - 0.77)	< 0.001	0.65	(0.58 - 0.73)	< 0.001
PFAA only	Ser	Thr	Cit	Lys	Ile	Trp	0.70	(0.64 - 0.77)	< 0.001	0.65	(0.58 - 0.73)	< 0.001
PFAA only	Ser	Asn	Thr	Cit	Lys	Trp	0.70	(0.64 - 0.77)	< 0.001	0.65	(0.57 - 0.73)	< 0.001

[1]The top 50 models based on the optimism-corrected AUC of ROC (training dataset) were selected for the validation process. The top 5 models with regard to validated performance are depicted. This model selection process was performed for "variables selected from Alb and PFAAs" and "variables selected from PFAAs only". [2]The p-values were obtained by performing a Mann–Whitney U-test between the MCI and CN groups.

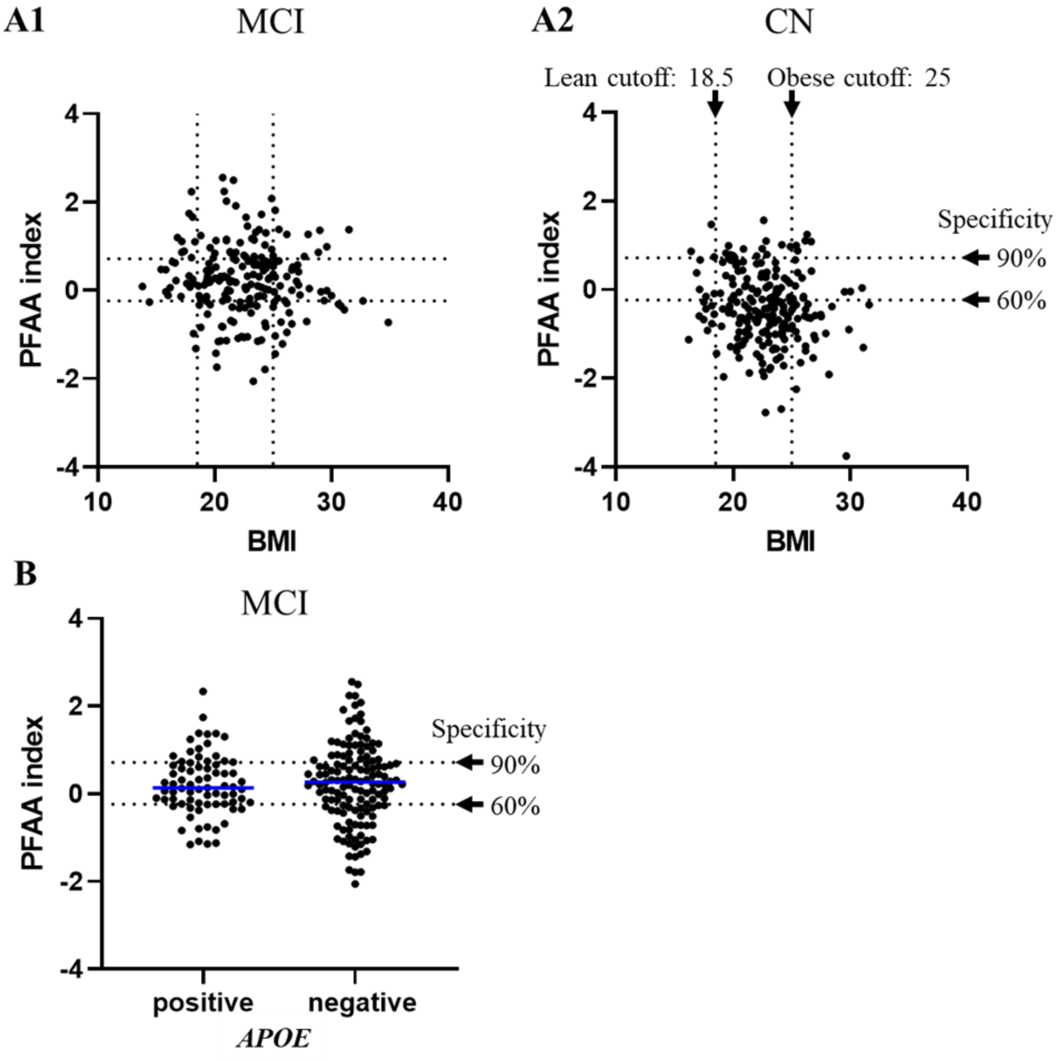

Figure S1. Relationships of the PFAA index with BMI and *APOE***.** Correlation of PFAA index values and BMI in MCI participants (n=200, r = -0.071, p = 0.316) (A1) and CN individuals (n=220, r = -0.169, p = 0.0122) (A2). The vertical dotted lines show the lean and obese cutoff values. PFAA index values according to *APOE* genotype (75 positive and 138 negative) in MCI (B). The blue lines indicate the median. There was no significant difference between the *APOE*-positive and *APOE*-negative groups (Mann–Whitney U-test p=0.7093). The horizontal dotted lines show the cutoffs with specificities of 90% and 60%.

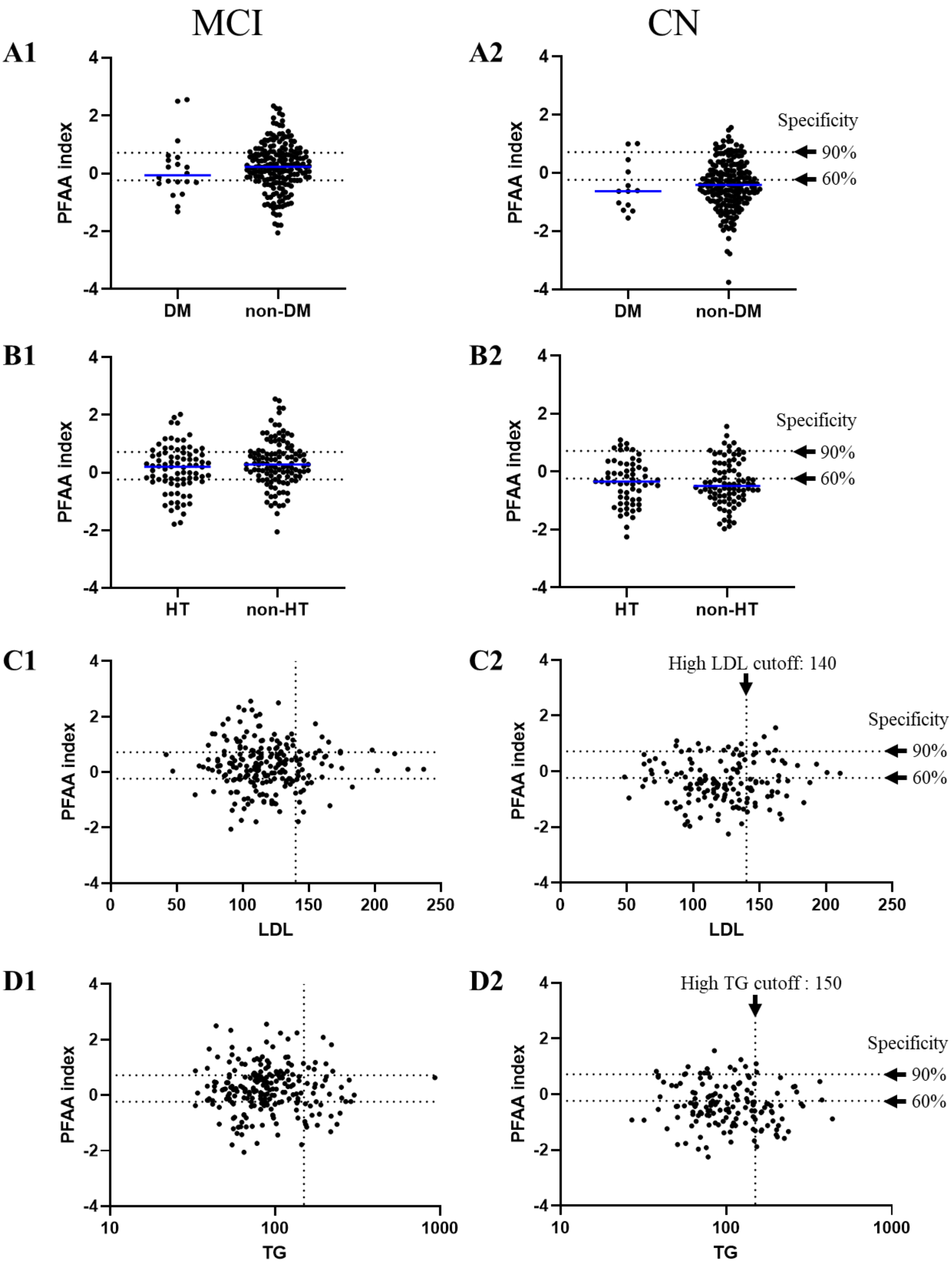

Figure S2. Relationships between PFAA index and lifestyle-related diseases The PFAA index values according to diabetes mellitus (DM) criteria in MCI participants (20 DM and 198 non-DM) (A1) and CN individuals (13 DM and 207 non-DM) (A2). PFAA index values according to hypertension (HT) criteria in MCI participants (82 HT and 117 non-HT) (B1) and CN individuals (61 HT and 86 non-HT) (B2). Correlation of PFAA index values and blood low-density lipoprotein cholesterol (LDL) levels in MCI participants ($n=219$, $r = -0.028$, $p = 0.683$) (C1) and CN individuals ($n=147$, $r = 0.017$, $p = 0.84$) (C2). Correlation of PFAA index values and blood triglyceride (TG) levels in MCI participants ($n=219$, $rs = 0.012$, $p = 0.866$) (D1) and CN individuals ($n=147$, $rs = -0.113$, $p = 0.174$) (D2). DM criteria were as follows: fasting blood glucose ≥ 126 [mg/dL] and/or HbA1c ≥ 6.5 [%]. HT criteria were as follows: systolic blood pressure ≥ 140 [mmHg] and/or diastolic blood pressure ≥ 90 [mmHg]. The horizontal dotted lines show the cutoffs with specificities of 90% and 60%. The vertical dotted lines show the cutoffs of high LDL and high TG. The blue lines show the median.
